# The Improvement Effects of *Weizmannia coagulans* BC99 on Liver Function and Gut Microbiota of Long-Term Alcohol Drinkers: A Randomized Double-Blind Clinical Trial

**DOI:** 10.3390/nu17020320

**Published:** 2025-01-17

**Authors:** Jie Zhang, Cheng Li, Mengyao Duan, Zhen Qu, Yi Wang, Yao Dong, Ying Wu, Shuguang Fang, Shaobin Gu

**Affiliations:** 1 College of Food and Bioengineering, Henan University of Science and Technology, Luoyang 471000, China; 2 Henan Engineering Research Center of Food Material, Henan University of Science and Technology, Luoyang 471023, China; 3 Department of Research and Development, Wecare Probiotics Co., Ltd., Suzhou 215200, China; 4 Germline Stem Cells and Microenvironment Lab, College of Animal Science and Technology, Nanjing Agricultural University, Nanjing 210095, China; 5 Henan Engineering Research Center of Food Microbiology, Luoyang 471000, China

**Keywords:** *Weizmannia coagulans* BC99, randomized controlled trial, alcoholic liver injury, gut microbiota, oxidative stress

## Abstract

Background/Objectives: With the improvement of living standards, alcoholic liver disease caused by long-term drinking has been a common multiple disease. Probiotic interventions may help mitigate liver damage caused by alcohol intake, but the mechanisms need more investigation. Methods: This study involved 70 long-term alcohol drinkers (18–65 years old, alcohol consumption ≥20 g/day, lasting for more than one year) who were randomly assigned to either the BC99 group or the placebo group. Two groups were given BC99 (3 g/day, 1 × 10^10^ CFU) or placebo (3 g/day) for 60 days, respectively. Before and after the intervention, blood routine indicators, liver function, renal function, inflammatory factors and intestinal flora were evaluated. Results: The results showed that intervention with *Weizmannia coagulans* BC99 reduced the levels of alanine aminotransferase, aspartate aminotransferase, glutamyl transpeptidase, serum total bilirubin, blood urea nitrogen, uric acid and ‘blood urea nitrogen/creatinine’. *Weizmannia coagulans* BC99 also reduced the levels of pro-inflammatory factors TNF-α and IL-6 and increased the levels of anti-inflammatory factor IL-10. The results of intestinal flora analysis showed that *Weizmannia coagulans* BC99 regulated the imbalance of intestinal flora, increased the beneficial bacteria abundance (*Prevotella*, *Faecalibacterium* and *Roseburia*) and reduced the conditionally pathogenic bacteria abundance (*Escherichia-Shigella* and *Klebsiella*). Both LEfSe analysis and random forest analysis indicated that the increase in the abundance of *Muribaculaceae* induced by BC99 was a key factor in alleviating alcohol-induced liver damage. Conclusions: These findings demonstrate that *Weizmannia coagulans* BC99 has the potential to alleviate alcoholic liver injury and provide an effective strategy for liver protection in long-term drinkers.

## 1. Introduction

Alcohol is one of the most commonly abused drinks by modern people. Alcoholic liver disease (ALD) caused by long-term drinking has been a common multiple disease [1]. In recent years, with the improvement of living standards, the drinking amount of residents has been increasing, and the incidence of ALD in China and most developed countries in Europe and America has been increasing year by year. According to the epidemiological investigation report [2], alcohol has become the second leading cause of liver damage after viral hepatitis. Globally, ALD causes more than 3.3 million deaths every year, accounting for 5.1% of the global disease burden [3]. The progress of ALD is mainly related to the amount and duration of alcohol consumption. The symptoms of patients are mostly progressive or occult, initially presenting as fatty liver, which can progress to alcoholic hepatitis, liver fibrosis and even cirrhosis [4]. Severe alcoholism can induce extensive hepatocyte necrosis and even lead to liver failure, which seriously endangers human life [5]. Modern medical research has proved that the liver is the main metabolic organ for ethanol, and long-term excessive alcohol consumption will lead to the accumulation of ethanol and acetaldehyde in the liver, which will lead to ALD through various mechanisms, such as damaging lipid metabolism, aggravating inflammatory reaction and inducing fibrosis [6]. At present, lifestyle change including abstinence from alcohol and a healthy diet is still one of the main treatments for ALD patients. However, because of the heavy dependence on alcohol, most long-term drinkers have difficulty sticking to lifestyle changes. Therefore, how to adopt effective methods to protect the liver from alcohol damage has become a new idea to reduce the occurrence of ALD.

There is a close relationship between liver metabolism and intestinal flora [7]. Gut microbiota can regulate liver metabolic function, which is of great significance for maintaining human health. Long-term excessive alcohol consumption will lead to significant changes in the composition of gut microbiota, especially by inhibiting the growth of beneficial bacteria and promoting the overgrowth of pathogenic bacteria in intestine, thereby disrupting the gut microbiota and damaging the intestinal barrier function [8,9]. Modern medicine believes that the etiology of ALD is clear, but the pathogenesis is complex, which has not been fully explained. The treatment still focuses on anti-inflammatory mechanisms and liver protection, but this method has significant side effects, and the therapeutic effect cannot satisfy patients.

As living microorganisms, probiotics are believed to improve gastrointestinal health, lower cholesterol and blood pressure, improve immune function and reduce inflammation [10]. Previous studies have discussed the use of probiotics to improve intestinal health, showing their potential to restore intestinal microflora and enhance intestinal–liver signal transduction. *Bifidobacterium* and *Lactobacillus* are widely regarded as typical probiotics, and their therapeutic effects on alcoholic liver injury have been extensively studied. *Bifidobacterium lactis* TY-S01 helps maintain the integrity of the intestinal barrier and the balance of the intestinal flora, thereby reducing alcohol-induced liver injury [11]. Wang et al. [12] found that *Lactobacillus rhamnosus* suspension (containing live bacteria, LGG) can effectively treat alcohol-induced liver fat accumulation and liver injury in mice. In recent years, there has been a growing interest in *Bacillus* as a probiotic due to its high tolerance to adverse environments. There are some studies on improving liver function through *Bacillus* intervention. Lu et al. [13] showed that *Bacillus subtilis* had protective effects on alcohol-induced liver injury in mice, indicating that probiotics also played a key role in alcoholism. *Weizmannia coagulans* BC99 probiotic has been reported to have the ability to relieve inflammation and oxidative stress, suggesting its potential in the treatment of alcoholic liver injury. Previous studies were carried out in vitro and in animals, but their effectiveness in relieving alcoholism and protecting the liver in the clinic has not been fully explored.

Therefore, in this study, a randomized, double-blind, placebo-controlled crossover study was conducted to evaluate the protective effects of *Weizmannia coagulans* BC99 on the livers of long-term drinkers. In addition to liver function indicators, the effects of *Weizmannia coagulans* BC99 on renal function, inflammatory factors and gut microbiota, and on the correlations among these characteristic indicators, were also be evaluated. This study can provide a more comprehensive scientific basis for the clinical application of *Weizmannia coagulans* BC99 in the prevention and treatment of alcoholic liver disease.

## 2. Materials and Methods

### 2.1. Study Subjects

A randomized, double-blind, placebo-controlled trial was conducted at Henan University of Science and Technology, Luoyang, China, from July 2024 to November 2024. The study protocol complies with the World Medical Association Declaration of Helsinki [14] and was approved by the Ethics Committee of the First Affiliated Hospital of Henan University of Science and Technology (NCT06607562, May, 2024). This study recruited 70 participants. All participants met the inclusion criteria: aged between 18–65 years old, with a long-term history of alcohol consumption (alcohol ≥ 20 g/day, lasting for more than 1 year, alcohol (g) = alcoholic beverage (mL) × alcohol content (%) × 0.8 (alcohol specific gravity)). People with basic diseases such as hepatitis, liver cirrhosis, hypertension and diabetes were not included in the recruitment. All participants were required to be informed of the research procedure and sign a written consent form before they could be included in the study.

### 2.2. Study Design

**Figure 1:**
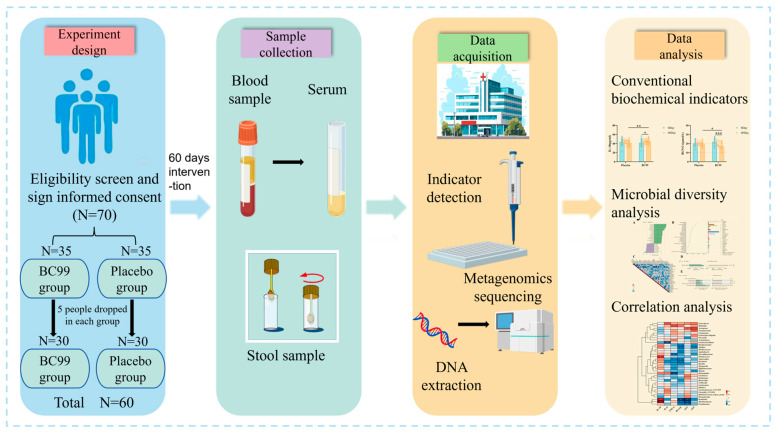
Flowchart of the study design.

A total of 72 participants were recruited for screening in this study, with 70 eligible participants. Among them, 60 participants completed the entire experimental process. As shown in Figure 1, 70 eligible participants were randomly assigned into a placebo group or a probiotic group (35 participants in each group). Participants in the placebo group were daily treated with placebo (composed of 3 g maltodextrin) intervention, while participants in the probiotic group were daily treated with *Weizmannia coagulans* BC99 (3 g, 1 × 10^10^ CFU) probiotic intervention. The total duration of the study was 60 days, and participants were followed up and sampled on day 1 and day 60, respectively. During the intervention period, participants in each group were required to take the corresponding products daily and record any adverse reactions. After the intervention period was completed, the remaining products and empty packaging boxes were all recovered.

### 2.3. Probiotic

*Weizmannia coagulans* BC99 was provided by Wecare Probiotics Co., Ltd., Suzhou, China. The entire development process of *Weizmannia coagulans* BC99 was carried out in an extremely professional production environment, with a 100,000 level GMP clean workshop and a constant temperature of 28 °C and humidity of 35%. At the same time, unique cell microencapsulation technology was used to ensure the stability and efficacy of *Weizmannia coagulans* BC99.

### 2.4. Collection and Determination of Blood Samples

Collection and biochemical determination of blood samples were conducted using clinical standard measurement methods after baseline abdominal blood collection and intervention for 60 days. Blood samples were collected for clinical chemical analysis after fasting for at least 10 h. Blood samples were centrifuged and the serum was stored at −80 °C before analysis. The liver and renal function indicators were measured using an automatic biochemical analyzer (KHB ZY-1280, Shanghai Kehua Biotechnology Co., Ltd., Shanghai, China). The inflammatory factor-related indicators including high-sensitive *C*-reactive protein (hs-CRP), tumor necrosis factor-α (TNF-α), interleukin-6 (IL-6) and interleukin-10 (IL-10) were measured using the commercial kit (Shanghai Hepeng Biotechnology Co., Ltd., Shanghai, China). The operation was carried out in strict accordance with the manufacturer’s protocol.

### 2.5. Collection and Preparation of Fecal Samples

After fasting for at least 10 h, fresh fecal samples were collected and stored sterile at −80 °C for analysis.

### 2.6. Intestinal Microbiota Analysis

The genomic DNA of the sample was extracted using the MagPureSoil DNA LQ Kit (Magan, Aurora, ON, Canada). The operation was carried out in strict accordance with the instructions. The concentration of the extracted DNA was determined using Ultramicrospectrophotometer NanoDrop 2000 (Thermo Fisher Scientific, Waltham, MA, USA). The purity of the extracted DNA was determined by agarose gel electrophoresis. The extracted genomic DNA was stored at −20 °C before analysis. The 16S rRNA gene was amplified using the universal primers 343F (primer sequence, 5′-TACGGRAGGCAGCAG-3′) and 798R (primer sequence, 5′-AGGGTATCTAATCCT-3′). PCR amplification products were determined by agarose gel electrophoresis. After two rounds of PCR amplification, the sequencing was performed using the Illumina NovaSeq6000 sequencing platform of Shanghai Ouyi Biotechnology Co., Ltd. (Shanghai, China). After quality control analysis of sequencing data, representative sequences and ASV abundance tables were obtained. The QIIME2 (2020.11) software package was used to select the representative sequences for each ASV. All representative sequences were compared against the Silva (version 138) database and then annotated. Species annotation was analyzed using the q2-feature-classifier (2020.11) software. Diversity analysis and differential analysis were performed using the QIIME2 software and the ANOVA *t*-test statistical algorithm based on the R package (3.5.1), respectively. Differential analysis of species abundance profiles was conducted using LEfSe (1.0.0).

### 2.7. Statistical Analysis

SPSS 25.0 was used for statistical analysis [15]. The experimental data are presented as mean ± standard deviation. The confidence interval is 95%. For intergroup comparison, if the data conform to the assumptions of normal distribution and homogeneity of variance, the independent sample *t*-test was used. If not, the Mann–Whitney U test was used. For multiple group comparison, if the data conform to the assumptions of normal distribution and homogeneity of variance, one-way ANOVA was used. If not, the Kruskal–Wallis test was used. *p* < 0.05 indicates a difference, *p* < 0.01 indicates a significant difference and *p* < 0.001 indicates an extremely significant difference. Graphpad Prism 10.1.2 (www.graphpad.com) software was used to draw graphics.

## 3. Results

### 3.1. Baseline Characteristics

The baseline characteristics of the participants such as mean age, sex distribution, metabolic characteristics, liver function indicators and renal function indicators are shown in Table 1. The mean age of the participants in the placebo group and the BC99 group was 43.60 ± 11.31 years and 42.87 ± 11.15 years, respectively. There was no significant difference in age and sex distribution between the placebo group and the BC99 group. The baseline BMI values, which were 24.24 ± 2.89 kg/m^2^ in placebo group and 24.87 ± 2.68 kg/m^2^ in BC99 group, also did not exhibit a significant difference between the two groups. Furthermore, no significant differences were observed in liver function indicators or renal function indicators between the two groups.

### 3.2. Blood Routine Indicators

The blood routine indicators of the placebo group and BC99 group before and after 60 days of intervention were compared, and the results were shown in Table 2. There was no significant difference in blood routine indicators between the placebo group and the BC99 group before and after 60 days of intervention (*p* > 0.05).

### 3.3. Liver Function Indicators

**Figure 2:**
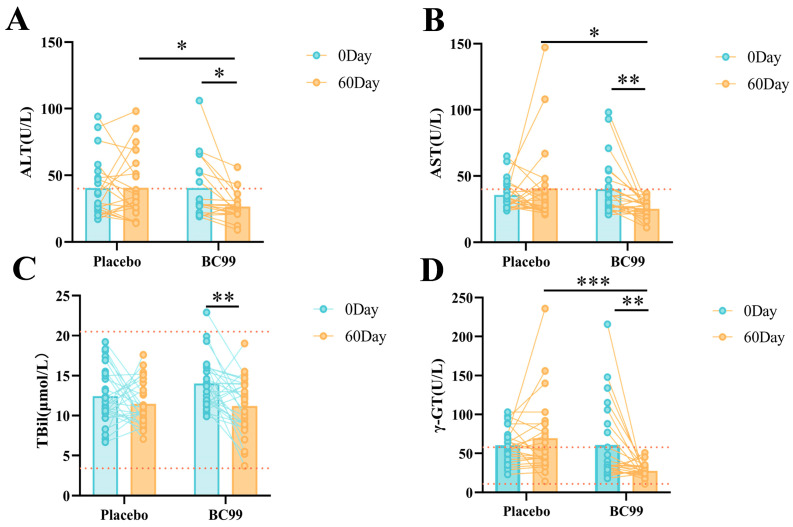
The regulatory effect of *Weizmannia coagulans* BC99 on liver function indicators. (**A**) The effect of BC99 on ALT. (**B**) The effect of BC99 on AST. (**C**) The effect of BC99 on TBil. (**D**) The effect of BC99 on γ-GT. * *p* < 0.05, ** *p* < 0.01, *** *p* < 0.001.

The differences in liver function indicators such as ALT, AST, TBil and γ-GT between the placebo group and the BC99 group after a 60-day intervention are shown in Figure 2. For the placebo group, there was no significant difference in the above indicators before and after the 60-day intervention. However, after 60 days of intervention in the BC99 group, three indicators, AST, TBil and γ-GT, showed significant decreases (*p* < 0.01, Figure 2B–D). Especially for AST and γ-GT, the unqualified rates of the two indicators were 34.78% and 30.43% before intervention, respectively, and all decreased to 0% after intervention. In addition, after 60 days of intervention, the ALT level in the BC99 group showed a decrease (Figure 2A), and the unqualified rates of the indicator decreased from 38.88% before intervention to 11.11% after intervention.

### 3.4. Renal Function Indicators

**Figure 3:**
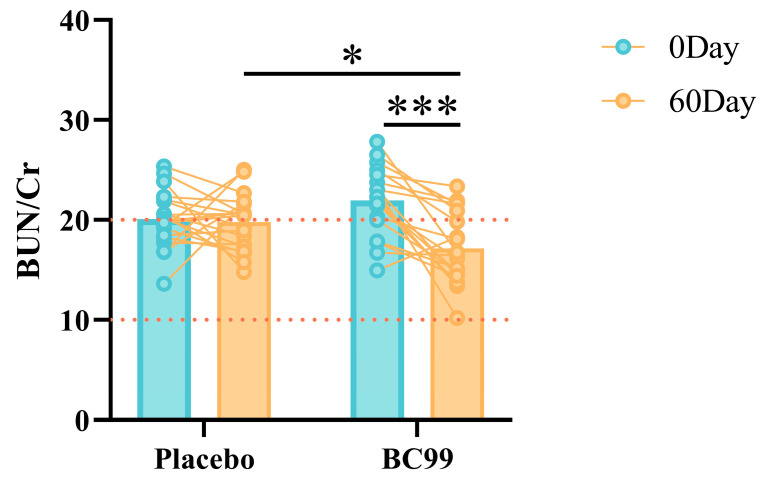
The regulatory effect of *Weizmannia coagulans* BC99 on “BUN (mg/dL)/Cr (mg/dL)”. The conversion relationship between different units refers to Beeler [16]. * *p* < 0.05, *** *p* < 0.001. The two red dashed lines represent the range of clinical normal values for this indicator.

The differences in renal function indicators such as BUN, UA and Cr between the placebo group and the BC99 group after a 60-day intervention are shown in Table 3. There was no significant difference in renal function indicators before and after the 60-day intervention for the placebo group. After 60 days of intervention in the BC99 group, two indicators, BUN and UA, showed a significant decrease, while the Cr level did not change significantly. However, as it is shown in Figure 3, the “BUN/Cr” level significantly decreased. In clinical practice, “BUN/Cr” is commonly used to characterize acute renal injury, indicating the degree of damage to glomerular filtration function. In this study, after 60 days of intervention, the number of subjects with abnormal “BUN/Cr” in the BC99 group decreased from 14 to five, while there was no significant difference in the placebo group.

### 3.5. hs-CRP and Inflammatory Factors

**Figure 4:**
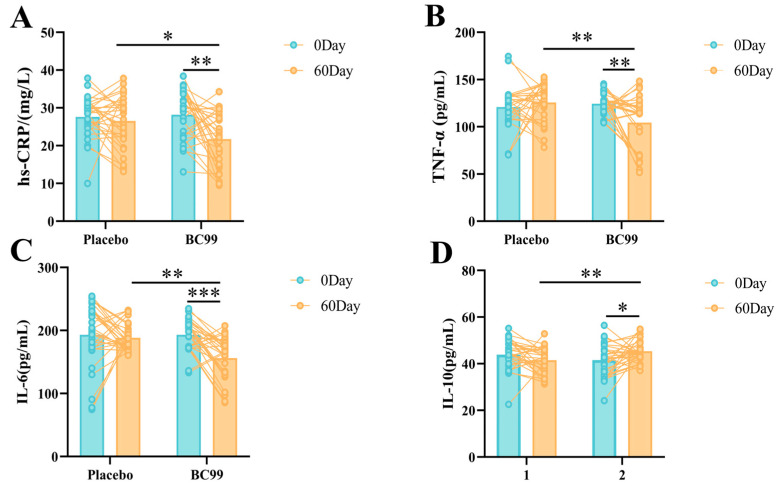
The regulatory effect of *Weizmannia coagulans* BC99 on hs-CRP (**A**) and inflammatory factors TNF-α (**B**), IL-6 (**C**) and IL-10 (**D**). * *p* < 0.05, ** *p* < 0.01, *** *p* < 0.001.

In this study, the hs-CRP levels before and after 60 days of intervention were compared between the placebo group and the BC99 group. As shown in Figure 4A, there was no significant difference in the hs-CRP levels of the placebo group before and after 60 days of intervention, while the BC99 group showed a significant decrease in hs-CRP levels after 60 days of intervention, from 28.18 mg/L to 21.71 mg/L. As for the pro-inflammatory factors TNF-α and IL-6, they also showed a similar trend of change (Figure 4B,C). There was no significant change in these two indicators before and after intervention in the placebo group. However, in the BC99 group, after 60 days of intervention, the levels of both indicators showed a significant downward trend, especially IL-6, which decreased from 193.08 mg/mL before intervention to 156.21 mg/mL after intervention, a decrease of more than 30%. In addition, 23 subjects showed a decreasing trend in the IL-6 level, accounting for 76.67% of the total participants. Furthermore, as can be seen from Figure 4D, the anti-inflammatory factor IL-10 level in the BC99 group was significantly increased after 60 days of intervention, from 41.55 pg/mL to 45.34 pg/mL. In contrast, the IL-10 level in the placebo group decreased slightly, from 43.92 pg/mL before intervention to 41.47 pg/mL after intervention.

### 3.6. Efficacy of BC99 on Gut Microbiota

**Figure 5:**
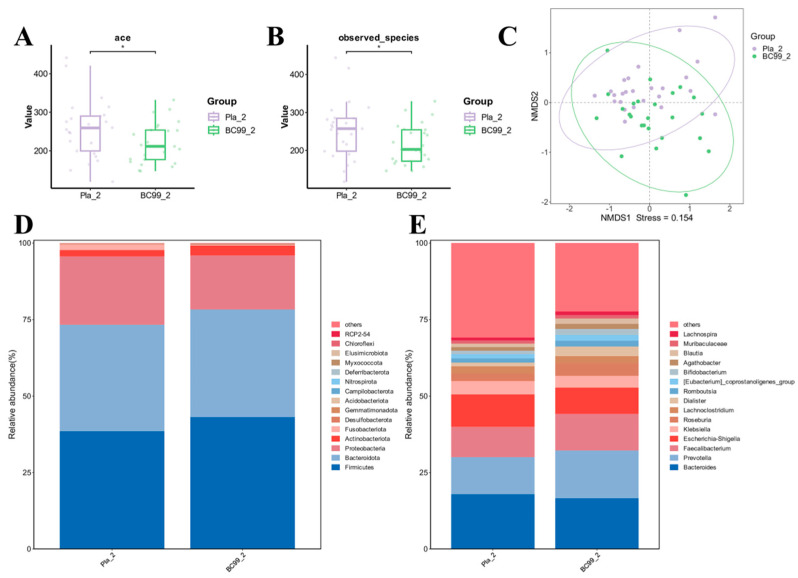
BC99 regulated the composition of gut microbiota. (**A**) ace indice; (**B**) observed indice; (**C**) NMDS analysis; (**D**) abundance of phylum-level flora; (**E**) abundance of genus-level flora. Pla_2 and BC99_2 represented samples from the placebo group and BC99 group after 60 days of intervention, respectively. * *p* < 0.05.

We further studied the effect of BC99 on the composition of the gut microbiota in long-term alcohol drinkers by using 16S rRNA gene sequencing of fecal samples. As shown in Figure 5A,B, the ace index evaluates the richness and evenness of species composition in the sample, and the observed species index indicates the number of species present in the sample. Compared with the placebo group, there were significant changes in the microbiota alpha diversity after intervention with BC99. Beta diversity is an indicator of the similarity in microbial community structure between samples. The diversity results based on NMDS analysis showed that the confidence ellipses between the two groups were not clearly separated, but some points of the BC99 group were distant from those of the placebo group, indicating that the intervention of BC99 had, to some extent, altered the gut microbiota structure (Figure 5C). The analysis of species structure adopts statistical methods and can obtain which dominant species are present in each sample at various levels (including domain, kingdom, phylum, class, order, family, genus, species, OTU, etc.), as well as the relative abundance of each dominant species. As shown in Figure 5D,E, the gut microbiota of the two groups included similar microbial community structures. At the phylum level, the gut microbiota was mainly composed of Firmicutes, Bacteroidota and Proteobacteria, accounting for over 90% of the human gut microbiota. Compared to the placebo group, the abundance of Firmicutes in the BC99 group increased while the abundance of Proteobacteria decreased. This indicated that the BC99 intervention significantly changed the gut microbiota at the phylum level in long-term alcohol drinkers. At the genus level, it was found that the most abundant genera in the gut microbiota of all groups were *Bacteroides*, *Prevotella*, *Faecalibacterium*, *Escherichia-Shigella*, *Klebsiella* and *Roseburia*. Compared to the placebo group, the BC99 group showed an increased abundance of *Prevotella*, *Faecalibacterium* and *Roseburia*, while the abundance of *Bacteroides*, *Escherichia-Shigella* and *Klebsiella* decreased.

**Figure 6:**
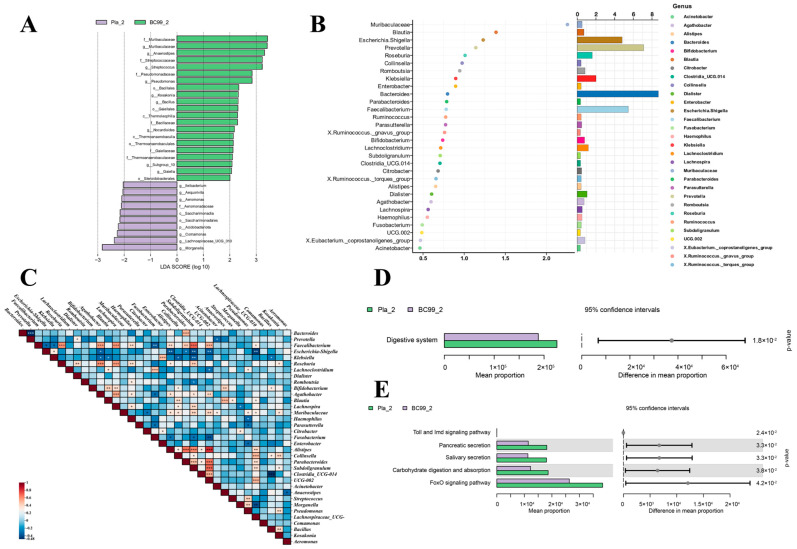
BC99 elevated the levels of beneficial bacteria while reducing the levels of pathogenic bacteria. (**A**) Analysis of differences in the microbial taxa by LEfSe; (**B**) random forest analysis; (**C**) analysis of flora interaction; (**D**) KEGG L2 metabolism pathway; (**E**) KEGG L3 metabolism pathway. Pla_2 and BC99_2 represented samples from the placebo group and BC99 group after 60 days of intervention, respectively. * *p* < 0.05, ** *p* < 0.01, *** *p* < 0.001.

According to LEfSe analysis (Figure 6A), differentially abundant microbial communities in the BC99 intervention can be identified. In the placebo group, the differential strains were found to be *Morganella*, *Lachnospiraceae* UCG-010 and *Comamonas*. The enrichment of *Muribaculaceae*, *Anaerostipes*, *Streptococcus*, *Pseudomnas* and *Kosakonia* were found in the BC99 group, and *Bacillus* was also detected in the BC99 group. These differential strains may be key strains in alleviating alcohol-induced damage in the BC99 group. To study the relationships among the microbiota, the present study conducted an analysis of microbial interactions (Figure 6C). The results showed that *Faecalibacterium* was significantly negatively correlated with *Escherichia-Shigella* and *Klebsiella*, and significantly positively correlated with *Roseburia* and *Lachnospiraceae* UCG-010. *Muribaculaceae* was significantly positively correlated with *Pseudomnas* and *Kosakonia*, while *Bacillus* was significantly negatively correlated with *Klebsiella*. It was also found that *Escherichia-Shigella* was significantly positively correlated with *Klebsiella*. This demonstrated that long-term alcohol consumption could lead to the mutual growth of conditionally pathogenic bacteria, while intervention with BC99 could synergistically increase the abundance of beneficial bacteria in the gut and suppress the growth of pathogenic bacteria. At the same time, random forest analysis was used to identify key species that could differentiate between groups. As shown in the Figure 6B, the most significantly important key strain was *Muribaculaceae*. This was also consistent with the conclusion of the LEfSe analysis, which indicated that *Muribaculaceae* may be the key strain for BC99 to alleviate alcohol damage.

Based on marker gene sequences, microbial community functional analysis was carried out using PICRUSt (2.3.0b0) software and the KEGG database. The results are shown in Figure 6D,E. At the KEGG L2 classification level, there was a significant difference in one pathway related to the digestive system between the two groups. Further screening at the KEGG L3 level revealed five different pathways, including the Toll and Imd signaling pathways and pancreatic secretion.

### 3.7. Correlation Analysis

**Figure 7:**
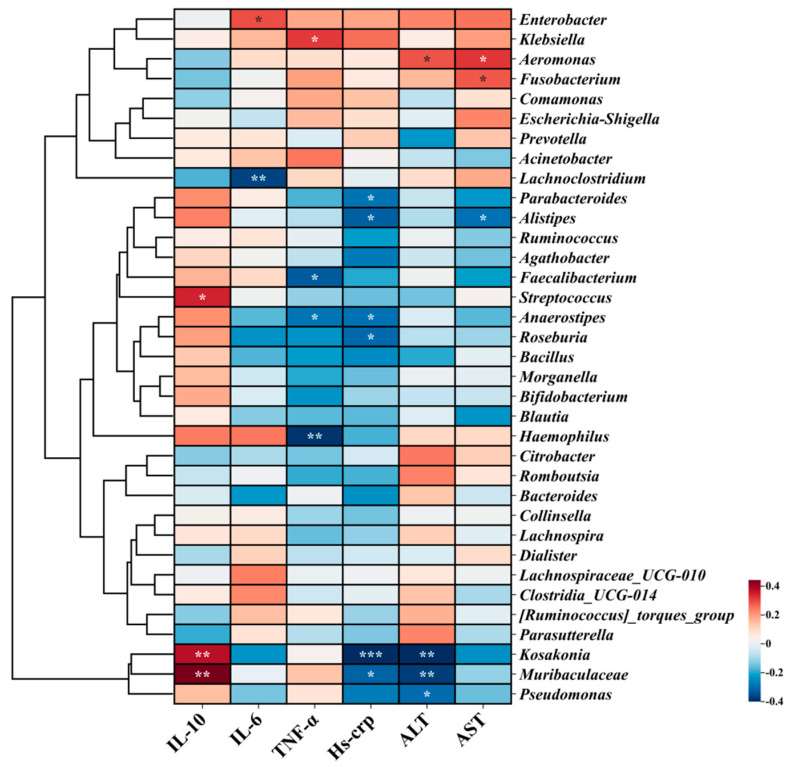
Correlation analysis of intestinal flora with inflammation and physiological indexes. * *p* < 0.05, ** *p* < 0.01, *** *p* < 0.001.

By performing correlation analysis between inflammatory factors and liver function indicators with the levels of intestinal microbiota, this study explored the key strains that alleviate liver damage in long-term drinkers. From Figure 7, it can be found that the differential strain *Klebsiella* in the placebo group was significantly positively correlated with the pro-inflammatory factor TNF-α. *Aeromonas* was significantly positively correlated with ALT and AST. This indicated a strong correlation between these strains and the body’s inflammatory response and liver damage. Additionally, the differential strain *Anaerostipes* in the BC99 group was significantly negatively correlated with TNF-α and hs-CRP. *Muribaculaceae* and *Kosakonia* were significantly positively correlated with anti-inflammatory factors IL-10 and significantly negatively correlated with hs-CRP and ALT, indicating that they may be the key strains in BC99 to alleviate acute alcohol poisoning.

## 4. Discussion

Drinking has become a popular form of social entertainment for people. According to the survey, the incidence rate of alcoholic liver disease is increasing year by year worldwide [17]. Long-term alcoholism will not only cause liver fibrosis and fatty liver, but can also lead to cirrhosis, liver cancer and other diseases, in severe cases, that seriously threaten the physical health of people [18]. The mechanism of action of alcoholic liver injury is related to a variety of factors, including oxidative stress reaction, inflammatory signaling pathways, etc. [19,20]. At present, clinical methods for treating alcoholic liver injury include drug-assisted therapy and alcohol abstinence, but the efficacy is not outstanding, and adverse reactions often occur after drug intervention [21]. A growing body of research suggests that using probiotics to control the gut microbiota may be a potential way to protect the liver from alcohol damage. In this work, we investigated the improvement effect of BC99 on the physical functions of long-term alcohol drinkers, mainly including liver function, renal function, inflammatory factors and gut microbiota. The final results indicated that BC99 indeed played a good role in the above aspects.

The liver, as the main organ for alcohol metabolism, plays a crucial role in its normal function. The results in this study indicated that after intervention with BC99, subjects’ liver function indicators including ALT, AST, TBil and γ-GT have significantly improved, demonstrating the good protective effect of BC99 on the liver. In addition, BC99 also significantly improved the renal function of the subjects, mainly reflected in the reduction in BUN, UA and BUN/Cr levels. Inflammatory factor analysis also confirmed the reliability of the above results. Hs-CRP is considered to be one of the most sensitive markers of inflammation. A large number of studies have shown that hs-CRP levels in alcohol-dependent patients were significantly higher than those in healthy individuals [22]. TNF-α is a pro-inflammatory cytokine mainly produced by macrophages and monocytes, which can lead to a series of problems such as hepatic steatosis, inflammatory cytokine infiltration and hepatocyte necrosis. Therefore, the reduction in the TNF-α level can effectively block the inflammatory pathway associated with liver injury and inhibit the development of liver disease [23]. As another typical pro-inflammatory factor, the release of IL-6 can induce local inflammatory responses in the body or liver [24]. Therefore, reducing the level of IL-6 can weaken local inflammation in the liver. As a typical anti-inflammatory factor, the effect of IL-10 is opposite to that of TNF-α and IL-6. IL-10 is an immunomodulatory cytokine produced primarily by Th2 cells and acts primarily through its receptors. It can not only down-regulate the inflammatory response by inhibiting the activation and proliferation of mononuclear macrophages, but also inhibit the synthesis and expression of inflammatory mediators IL-1, IL-6, IL-8 and TNF in mononuclear macrophages in a broad spectrum [25,26]. The occurrence of inflammatory response is usually closely related to the oxidative stress of the body. Animal experiments have shown that *Weizmannia coagulans* BC99 can increase the levels of superoxide dismutase (SOD) and glutathione (GSH), thereby enhancing the body’s antioxidant capacity [27]. On one hand, this helps to resist oxidative stress caused by alcohol metabolism, and on the other hand, it can reduce the toxicity of drugs and chemicals, alleviate liver damage and slow down the progression of liver diseases [28].

In order to explore the mechanism by which *Weizmannia coagulans* BC99 alleviates liver damage in long-term drinkers, we further analyzed the gut microbiota. Through the analysis of microbial diversity, it was found that BC99 significantly altered microbial diversity, but the samples of the BC99 group were not completely separated from those of the placebo group, which may be related to factors such as intervention dosage and intervention time. Long-term alcohol consumption leads to a significant decrease in the relative abundance of the Firmicutes phylum [29] and a significant increase in the relative abundance of the Bacteroidetes phylum [30] in the gut, which is consistent with the results obtained in this study. Compared with the placebo group, the relative abundance of the Firmicutes phylum significantly increased in the BC99 group, while the relative abundance of the Bacteroidetes phylum decreased. At the taxonomic level, BC99 intervention reduced the relative abundance of *Escherichia-Shigella* and *Klebsiella*. In patients with alcoholic liver cirrhosis, the relative abundance of *Escherichia-Shigella* significantly increases and is associated with liver inflammation and fibrosis [31]. *Klebsiella*, a gram-negative bacterium, with increased abundance, can lead to liver cell damage and may further exacerbate liver injury by activating immune responses (such as the IFN-γ/STAT/IRF-1 signaling pathway) and the expression of inflammatory factors (such as TNF-α, IL-6) [32]. Meanwhile, intervention with BC99 increased the relative abundance of *Prevotella*, *Faecalibacterium* and *Roseburia*. Prevotella belongs to the Bacteroidetes phylum, and studies have shown a significantly decreased relative abundance in alcohol-induced liver injury mice [33], which is consistent with our clinical findings. In addition to assisting in the breakdown of proteins and carbohydrates, *Prevotella* can also produce short-chain fatty acids (SCFAs) [34]. SCFAs such as acetate and butyrate can protect the liver by enhancing intestinal barrier function. *Faecalibacterium* is a symbiotic bacterium that is believed to have anti-inflammatory properties and is an important producer of butyric acid, playing a significant role in the gut microbiota. The abundance of *Faecalibacterium* is usually reduced in ALD patients [35], consistent with the results of this study. *Roseburia*, through the production of SCFAs such as butyric acid, can regulate immune responses and inflammation. Studies have shown that supplementation of bacteria belonging to the *Roseburia* genus can improve alcohol-induced fatty liver in mice [36]. Lefse analysis was performed to analyze the key strains of bacteria responsible for alcohol-induced damage and the alleviation of damage by BC99. As shown in the Figure 6A, the most significant differential strain in the placebo group was *Morganella*. *Morganella* is a Gram-negative opportunistic pathogenic bacterium that is commonly found in the intestines of humans and other animals. It is an opportunistic pathogen that can cause various infections and induce a variety of diseases, including sepsis, liver abscess and central nervous system infections [37] when the host’s immune system is compromised.

In the BC99 group, the most significant differential strain was *Muribaculaceae*. *Muribaculaceae* is a bacterial family in the order Bacteroidales. *Muribaculaceae* can produce SCFAs through endogenous (mucin glycans) and exogenous polysaccharides (dietary fiber) [38]. It is worth mentioning that more and more plant extracts such as polysaccharides and polyphenols have been proven to have health promoting effects. Zhang et al. found that *Artemisia argyi* polysaccharides can induce the body to produce a large amount of SCFAs to alleviate intestinal inflammation and gut microbiota imbalance [39]. Flavonoid-rich mulberry leaf extract has been reported to regulate lipid metabolism and intestinal microbiota disorders caused by a high-fat diet [40]. Therefore, combining probiotics with some plant supplements may achieve better effects [41]. In addition, the abundance of *Muribaculaceae* is negatively correlated with inflammatory factors in the serum, and it can inhibit the activation of CD8+ T cells, resisting immune stimulation, indicating its potential anti-inflammatory and immune-regulating functions [42]. The study of Zhang [43] can also prove that *Muribaculaceae* may have an anti-inflammatory effect, and thus, it has a certain protective effect against liver injury caused by alcohol. Furthermore, the key strain screened by the random forest analysis method was *Muribaculaceae*. Therefore, we have reason to speculate that *Muribaculaceae* may be the key strain in BC99 alleviating liver damage in long-term alcohol drinkers. The analysis of microbial interactions showed that beneficial bacteria and pathogenic bacteria have an antagonistic relationship. BC99 intervention increased the abundance of beneficial bacteria and reduced the abundance of pathogenic bacteria. BC99 intervention can reduce alcohol-induced liver injury by regulating intestinal flora structure and restoring microbial imbalance caused by long-term alcohol consumption.

By conducting PICRUSt functional prediction analysis on various microbial groups, it was found that the pathways at the KEGG L2 level were related to the digestive system. Alcohol can cause direct damage to the digestive system during its absorption process in the human body. Alcohol can increase intestinal permeability, leading to dysbiosis of the gut microbiota and the release of bacterial products, exacerbating inflammation in the body [27]. At the KEGG L3 level, the Toll and Imd signaling pathways mainly involve the activation of the innate immune system and the regulation of inflammatory responses. According to reports, TLR4 can promote the production of inflammatory factors (such as TNF-α and IL-1β) and affect the activation of hepatic stellate cells, thus exacerbating liver fibrosis and inflammatory reactions in alcohol-induced liver injury by activating Kupffer cells [44]. Long-term alcohol consumption can lead to excessive stimulation and increased secretion of pancreatic exocrine function. In addition, alcohol can also affect the response of pancreatic cells to cholecystokinin (CCK) and other stimuli, leading to abnormal pancreatic secretion function [45]. The specific role of carbohydrate digestion and absorption in alcoholic liver injury may be related to its impact on liver metabolism. For example, excessive intake of carbohydrates may lead to insulin resistance and endoplasmic reticulum stress, both of which are associated with an increased risk of alcoholic liver injury [46]. The FoxO signaling pathway plays a key role in oxidative stress and cell apoptosis. Among the FoxO family, FoxO3a, as a mitochondrial protein, regulates the levels of SOD messenger RNA and protein in response to oxidative stress, thereby maintaining mitochondrial homeostasis [47]. Based on the literature and previous research, it is speculated that BC99 may improve the digestive system and alleviate alcohol-induced damage by regulating the occurrence of inflammatory oxidative stress. This provides a basis for further exploration of the mechanism of action of BC99 in the treatment of alcohol-induced liver injury.

This study suggests that BC99 can alleviate alcoholic liver injury in long-term alcohol drinkers, and is more comprehensive compared to previous research. In addition to evaluating the improvement of liver function indicators through BC99 intervention, it also elaborated on regulating inflammatory factors, alleviating oxidative stress and regulating gut microbiota. However, additional research is needed to uncover which microbial groups or key metabolites are playing a role.

## 5. Conclusions

In summary, the findings in this study demonstrate that BC99 intervention can effectively improve liver function (ALT, AST, TBil and γ-GT) and renal function (BUN, UA, Cr and BUN/Cr) in long-term alcohol drinkers, and that it also has a good regulatory effect on inflammatory factors (hs-CRP, TNF-α, IL-6 and IL-10). The potential mechanism by which BC99 effectively regulates clinical indicators may be attributed to its impact on key gut microbiota genera (*Muribaculaceae*, *Prevotella*, *Faecalibacterium*, *Escherichia-Shigella*, *Klebsiella* and *Roseburia*). These results will provide scientific evidence that BC99 intervention can reduce the extent of damage to the liver caused by alcohol intake. Future research may focus on exploring the deeper mechanisms by which BC99 affects these key bacterial genera or metabolites.

## Figures and Tables

**Table 1:** Baseline characteristics in the placebo group and BC99 group.

Projects	Placebo Group (*n* = 30)		BC99 Group (*n* = 30)		*p* Value
	Mean	SD	Mean	SD	
Age	43.60	11.31	42.87	11.15	0.801
Sex (M/F)	30/0		29/1		0.313
Metabolic characteristics					
Height (cm)	173.4	5.02	171.03	5.38	0.084
Weight (kg)	72.85	9.15	72.63	7.09	0.919
BMI (kg/m^2^)	24.24	2.89	24.87	2.68	0.382
Liver function indicators					
ALT (U/L)	40.60	22.81	40.50	23.73	0.990
AST (U/L)	35.82	11.13	40.17	21.38	0.392
γ-GT (U/L)	60.79	22.49	61.08	51.06	0.979
TBil (μmol/L)	12.42	3.48	14.03	3.11	0.073
Renal function indicators					
BUN (mmol/L)	5.92	0.88	6.16	0.78	0.393
Cr (μmol/L)	73.67	10.20	70.41	10.20	0.325
UA (μmol/L)	393.67	75.41	406.74	71.13	0.500

Abbreviations: ALT, alanine aminotransferase; AST, aspartate aminotransferase; γ-GT, glutamyl transpeptidase; TBil, serum total bilirubin; BUN, blood urea nitrogen; Cr, creatinine; UA, uric acid.

**Table 2:** Comparison of blood routine indicators before and after intervention.

Projects	Placebo Group (*n* = 30)			BC99 Group (*n* = 30)		
	0 Day	60 Day	*p-*Value	0 Day	60 Day	*p-*Value
Neutrophils (×10^9^/L)	3.08 ± 1.15	3.44 ± 1.42	0.277	3.04 ± 0.77	3.2 ± 0.75	0.429
Lymphocyte (×10^9^/L)	1.99 ± 0.51	2.08 ± 0.66	0.558	1.99 ± 0.53	3.36 ± 5.94	0.213
Red blood cell (×10^9^/L)	4.73 ± 0.80	4.48 ± 0.32	0.122	4.48 ± 0.42	4.36 ± 0.37	0.266
Hemoglobin (g/L)	136.83 ± 13.73	130.93 ± 11.06	0.072	141.67 ± 35.74	132.83 ± 10.90	0.201
Platelet (×10^9^/L)	181.33 ± 52.43	170.40 ± 46.44	0.396	179.53 ± 50.24	172.16 ± 68.50	0.637

**Table 3:** Comparison of renal function indicators before and after intervention.

Projects	Placebo Group (*n* = 30)			BC99 Group (*n* = 30)		
	0 Day	60 Day	*p-*Value	0 Day	60 Day	*p-*Value
BUN (mmol/L)	5.92 ± 0.88	5.67 ± 0.87	0.366	6.16 ± 0.78	5.00 ± 0.98	<0.001
UA (μmol/L)	393.67 ± 75.41	375.11 ± 113.17	0.466	406.74 ± 71.13	365.99 ± 61.68	0.023
Cr (μmol/L)	73.67 ± 10.20	73.25 ± 11.04	0.902	70.41 ± 10.20	72.91 ± 9.12	0.431

Abbreviations: BUN, blood urea nitrogen; UA, uric acid; Cr, creatinine.

## Data Availability

The original contributions presented in the study are included in the article; further inquiries can be directed to the corresponding author.
